# Intergenerational Sublethal Effects of Flonicamid on Cotton Aphid, *Aphis gossypii*: An Age-Stage, Two-Sex Life Table Study

**DOI:** 10.3390/insects15070529

**Published:** 2024-07-13

**Authors:** Hina Gul, Ali Güncan, Farman Ullah, Nicolas Desneux, Xiaoxia Liu

**Affiliations:** 1 MARA Key Laboratory of Pest Monitoring and Green Management, Department of Entomology, College of Plant Protection, China Agricultural University, Beijing 100193, China; gulhina680@gmail.com; 2 Department of Plant Protection, Faculty of Agriculture, Ordu University, 52200 Ordu, Turkey; guncan.ali@gmail.com; 3 State Key Laboratory for Managing Biotic and Chemical Threats to the Quality and Safety of Agro-Products, Institute of Plant Protection and Microbiology, Zhejiang Academy of Agricultural Sciences, Hangzhou 310021, China; farmanullah787@gmail.com; 4 Université Côte d’Azur, INRAE, CNRS, UMR ISA, 06000 Nice, France

**Keywords:** aphids, sublethal effects, insecticide toxicity, life table, demographic parameters

## Abstract

**Simple Summary:**

*Aphis gossypii* Glover is an economically important sap-sucking insect pest that causes severe damage worldwide. The flonicamid has been widely used for controlling sap-sucking insect pests, but its intergenerational sublethal effects on key demographic parameters of *A. gossypii* have not been fully studied. The age-stage, two-sex life table analysis was conducted to investigate the sublethal effects of flonicamid on the biological parameters of adult *A. gossypii* (F_0_) and its subsequent intergenerational effects on the offspring (F_1_ generation). The results showed that the sublethal concentrations of flonicamid significantly decreased the longevity, fecundity, and reproductive days of F_0_ *A. gossypii*. Moreover, flonicamid induced intergenerational sublethal effects on the subsequent progeny generation (F_1_) by impacting the key biological and demographic parameters of *A. gossypii*. Taken together, these results demonstrated that sublethal concentrations of flonicamid negatively affect the demographic parameters of *A. gossypii*, resulting in the suppression of population growth.

**Abstract:**

Flonicamid is a novel systemic insecticide widely used against aphids. However, the intergenerational sublethal effects of flonicamid on cotton aphid, *Aphis gossypii,* have not been fully studied. This study aimed to evaluate the sublethal effects of flonicamid on the biological parameters of adult *A. gossypii* (F_0_) and its subsequent intergenerational effects on the offspring (F_1_ generation) through age-stage, two-sex life table analysis. The results of the bioassays indicate that flonicamid exhibits significant toxicity toward adult *A. gossypii*, as evidenced by an LC_50_ value of 0.372 mg L^−1^ after a 48-h exposure period. The longevity, fecundity, and reproductive days of adult cotton aphids (F_0_) were significantly decreased when treated with the sublethal concentrations of flonicamid. The pre-adult stage exhibited an increase, whereas the adult longevity, total longevity, and fecundity experienced a notable decrease in F_1_ aphids after the exposure of F_0_ aphids to sublethal concentrations of flonicamid. Furthermore, the key demographic parameters, including *r*, *λ*, *R*_0_, and RP_d_, showed a significant decrease, while the total pre-reproductive period (TPRP) experienced a significant increase in the F_1_ generation. Collectively, our findings indicate that sublethal concentrations of flonicamid impact the demographic parameters of *A. gossypii*, resulting in suppression of population growth. This study presents comprehensive information on the overall impact of flonicamid on *A. gossypii*, which could potentially aid in managing this major pest.

## 1. Introduction

The cotton aphid, *Aphis gossypii* Glover (Hemiptera: Aphididae), is a serious sap-sucking insect pest that incurs substantial financial costs globally. *Aphis gossypii* inflicts severe agricultural damage through direct feeding, as well as indirect damage through virus transmission and honeydew contamination [1]. Several alternative pest management options are available [2,3,4,5], but insecticide application is still considered a major option in IPM [6,7]. Flonicamid is a recently developed systemic insecticide that poses a significant threat to sap-sucking insect pests [8,9,10]. Flonicamid exhibits high efficacy against phloem-feeding insects, including white flies, mealybugs, leafhoppers, thrips, jassids, scales, and aphids, by impeding their ability to engage in feeding behavior in the immediate aftermath of treatment [11]. Recently, Gul et al. (2023) reported that sublethal concentrations of flonicamid significantly affected key life-history traits on *Schizaphis graminum* (Rondani) (Hemiptera: Aphididae) [12]. The sublethal concentrations of flonicamid decreased the fecundity of *Myzus persicae* (Sulzer) (Hemiptera: Aphididae) [13]. The sublethal and low lethal concentrations of flonicamid significantly reduced the longevity and fecundity as well as the mRNA expression levels of genes related to the development and reproduction of *A. gossypii* [8]. Furthermore, Ren et al. (2018) revealed that sublethal concentrations of flonicamid inhibited the activity of *Kir1* channels and influenced the secretion of honeydew and saliva in *Nilaparvata lugens* (Stål) (Hempitera: Delphacidae) [10].

Agroecosystems may subject insects to sublethal concentrations of chemical insecticides due to biotic or abiotic factors contributing to incorrect use or degradation [14,15]. Arthropods exposed to chemical insecticide residues experience sublethal effects [16,17]. Insects and their subsequent generations experience a direct impact on life-history parameters due to these sublethal effects [8,18,19,20]. The sublethal effects of insecticides are determined by several aspects, including dose/concentration [21,22]. The metabolic activities of exposed individuals are stimulated by insecticide doses and concentrations, which affect the insect growth parameters [23]. Hormesis phenomena refer to the occurrence of stimulation at low doses or concentrations and inhibition at higher doses or concentrations [24]. As a result, sublethal effects are essential to determine whether insecticides have an overall impact on target or non-target pests [25]. Insecticides have sublethal effects on the physiological and behavioral characteristics of the directly exposed insects, including feeding activity, lifespan, developmental period, and fertility, as opposed to their lethal effects [26,27,28,29]. These intergenerational sublethal effects influence subsequent generations, ultimately leading to community alterations and ecological services [30,31]. The age-stage, two-sex life table approach is widely used to investigate the life-history traits of insects after exposure to different biotic and abiotic constraints [32,33,34,35]. This approach has been broadly used for investigating the lethal, sublethal, and intergenerational effects of insecticides on insects [12,36,37].

In the current study, we aimed to determine the acute toxicity of flonicamid in adult *A. gossypii*. In addition, we used age-stage, two-sex life table analysis to investigate the sublethal effects of flonicamid on directly exposed F_0_ generation aphids and to assess its subsequent intergenerational effects on the F_1_ generation of *A. gossypii*. These findings provide in-depth knowledge about the overall impact of flonicamid on *A. gossypii*, which could help to control this major pest.

## 2. Material and Methods

### 2.1. Insect and Insecticide

The population of *A. gossypii* was collected from Xinjiang Uygur Autonomous Region of China in 1999 and was reared in the laboratory (22 ± 1 °C, 70 ± 10% R.H., 16:8 L:D) for more than 15 years without exposure to any insecticides. Insecticide-free fresh seedlings of the cotton plant, *Gossypium hirsutum* (L.) were used to rear *A. gossypii*. First, the seedlings were washed and put in plastic cups filled with water. The plastic cups were placed in a cage, and the aphids were carefully transferred with a soft brush. All experiments were conducted under laboratory conditions with 22 ± 1 °C, 70 ± 10% R.H, and 16:8 L:D.

### 2.2. Bioassays

Flonicamid toxicity against *A. gossypii* was determined by a previously described leaf-dipping approach [38] with slight modifications [18,39]. To evaluate the toxicity, the corresponding stock solution (highest concentration) was used to prepare six different concentrations of flonicamid. The cotton leaves were carefully divided into circular discs of 20 mm in diameter using a pointed steel punch. The cotton leaf discs were immersed in either flonicamid (2, 1, 0.5, 0.25, 0.125, and 0.0625 mg L^−1^) or distilled water (control) for 15 s. The treated leaf discs were placed on disposable polyethylene gloves at room temperature to dry naturally. The dried leaf discs were placed on 2% agar in 12-well cell plates. Adult *A. gossypii* (wingless) were transferred to leaf discs with a soft brush. The plates were covered with Chinese art paper, specifically Xuan paper, to prevent aphids from escaping. A minimum of 30 aphids were used in each replicate, and the procedure was replicated thrice for every concentration. The mortality rate was assessed 48 h after flonicamid exposure. The aphids were deemed dead if they exhibited immobility with gentle manipulation using a soft brush.

### 2.3. Sublethal Effects of Flonicamid on Life-History Traits of the F_0_ Generation

A total of 300 apterous adult cotton aphids were placed onto newly grown cotton plants. Adult aphids were taken out after 24 h, while the newly emerged nymphs were kept for further maturation into apterous adults. This approach ensures the same developmental stage of all aphids when first treated with the flonicamid and was considered as the F_0_ generation. Flonicamid was diluted with distilled water to make the LC_5_ (0.077 mg/L) and LC_10_ (0.109 mg/L) concentrations. Cotton leaf discs (20 mm) were subjected to a 15-s immersion in solutions containing different concentrations of flonicamid (LC_5_ and LC_10_), or in distilled water (control). Subsequently, each treated leaf disc was placed onto a plastic sheet and allowed to air at room temperature for drying. The desiccated leaf discs were transferred to agar within the 12-well cell-culture plates. The wingless adult aphids were then carefully transferred onto the cotton leaf discs and properly covered with filter paper to prevent any possibility of escape. The cell plates were placed within the incubator. After a 48-h duration, a total of 40 aphids that were still alive were individually relocated from each experimental group (LC_5_, LC_10_, and control) onto fresh cotton leaf discs measuring 20 mm in diameter without using insecticides. During the experiment, new insecticide-free cotton leaf discs were replaced every 2–3 days. The observation of development, survival, and fecundity was conducted daily. Nymphs that had just been born were counted daily and taken away until adult aphids died.

### 2.4. Intergenerational Effects of Flonicamid on the Biological Traits of F_1_ A. gossypii

The biological parameters of the progeny generation (F_1_) were investigated whose parents were subjected to LC_5_ and LC_10_ of flonicamid. The experimental procedure was performed on 40 newly born F_1_ offspring for the flonicamid treatments and the control group. Daily monitoring was conducted to determine the life table parameters such as development, fecundity, and longevity of the F_1_ aphids. After counting, the newly born nymphs were removed daily to ensure only adults remained in the cells. Insecticide-free cotton leaf discs were changed every two to three days until the adult aphids died. Developmental durations, longevity, and fecundity were assessed for each aphid throughout the experiment.

### 2.5. Statistical and Life Table Analysis

The bioassay data were analyzed by PoloPlus v2 (LeOra Software Inc., Berkeley, CA, USA). The life table data of all cohorts were analyzed using an age-stage, two-sex life table technique [33,40]. The age-stage-specific survival rate (*s_xj_*), the age-specific survival rate (*l_x_*), the age-stage reproductive value (*v_xj_*), as well as life history traits like fecundity (*F*) (nymphs/female), reproductive days, adult longevity, development time, the adult pre-reproductive period (APRP), the total pre-reproductive period (TPRP), the intrinsic rate of increase (*r*), the finite rate of increase (*λ*), the net reproductive rate (*R*_0_), and the mean generation time (*T*) were assessed by a TWOSEX-MS Chart computer program [33,41]. The differences and standard errors (SEs) were investigated using 100,000 bootstrap replicates [42,43,44,45]. A paired bootstrap test (5% significant level) was used to assess the disparities in biological parameters between the flonicamid-exposed group and the control group on the basis of the confidence interval of the difference [46]. The detailed equations used for the life table analysis are shown in the Supplementary Materials File.

### 2.6. Population Projection

The population projections of different cohorts of *A. gossypii* were determined by the TIMING-MSChart program [47], according to the methodology described by [48]. The initial aphid population comprised ten newly born individuals in each cohort (control, LC_5_, and LC_10_). The populations were projected for 50 days, assuming no suppression by biotic or abiotic factors. To assess projection uncertainty, we conducted 100,000 bootstrap iterations and organized the resulting values of the net reproductive rate (*R*_0_). Then, the 2.5th and 97.5th percentiles, corresponding to the 2500th and 97,500th sorted bootstrap samples, were identified. The bootstrap life table samples were used to project the population for 50 days, resulting in these *R*_0_ values at the 2.5th and 97.5th percentiles. These findings underscored the uncertainty of the projected populations, obtaining confidence intervals of the projections [49].

## 3. Results

### 3.1. Toxicity of Flonicamid on Adult Aphis gossypii

The objective of this study was to assess the toxicity of flonicamid in adult cotton aphids using a leaf-dip bioassay procedure. The experimental findings indicated the high toxicity of flonicamid against adult *A. gossypii* following 48 h of treatment. The lethal concentrations that cause 50% mortality (LC_50_) were determined to be 0.372 mg L^−1^ (0.318–0.437 mg L^−1^) with slope ± SE of 2.403 ± 0.205 (*χ*^2^ = 8.826, *df* = 16, *p* = 0.920). The LC_5_ and LC_10_ values of flonicamid against adult cotton aphids were 0.077 mg L^−1^ (0.054–0.100 mg L^−1^), and 0.109 mg L^−1^ (0.081–0.136 mg L^−1^), respectively. The LC_5_ and LC_10_ were chosen to explore the direct effects of flonicamid on parental aphids as well as the indirect effects (intergenerational) on the biological and demographic parameters of progeny generation *A. gossypii*.

### 3.2. Sublethal Effects of Flonicamid on Parental Aphis gossypii (F_0_)

The adult longevity and fecundity of *Aphis gossypii* were significantly affected by the LC_5_ and LC_10_ of flonicamid (Table 1). A significant reduction in the longevity of *A. gossypii* was observed following exposure to flonicamid concentrations (LC_5_ and LC_10_), compared to the control group (*p* < 0.05). Adult cotton aphids subjected to LC_5_ and LC_10_ treatments exhibited a notable reduction in fecundity compared to the untreated control group (*p* < 0.05). The flonicamid-treated LC_5_ and LC_10_ individuals had fewer reproductive days than those in the control group (Table 1, *p* < 0.05).

### 3.3. Duration of Development and Adult Longevity of F_1_ Aphis gossypii

The intergenerational sublethal effects on F_1_ aphids whose parents were subjected to flonicamid LC_5_ and LC_10_ concentrations are presented in Table 2. The findings showed that exposure to flonicamid at LC_10_ concentration resulted in a slight prolongation of the developmental period of the 1st instar compared to the control group (Table 2, *p* < 0.05). However, there were no significant differences in the duration of 2nd and 3rd instar aphids at the LC_5_ and LC_10_ concentrations compared to the control (*p* < 0.05). The duration of the 4th instar and preadult period of *A. gossypii* increased statistically in the LC_10_ group, followed by the LC_5_ (*p* < 0.05). As a result, adult longevity and total longevity of F_1_ aphids decreased substantially at the LC_5_ and LC_10_ of flonicamid when the parental aphids were subjected to both concentrations compared to the control group (Table 2).

### 3.4. Reproduction and Life Table Parameters of F_1_ Aphis gossypii

The reproduction and life-history traits of progeny generation (F1) are shown in Table 3. The results demonstrated that *R*_0_, fecundity, and reproductive days (RP_d_) of F_1_ aphids were substantially decreased (*p* < 0.05) at the LC_10_ followed by the LC_5_ of flonicamid. The *r* and *λ* were substantially reduced at LC_5_ and LC_10_ concentrations. In contrast, there was no statistically significant difference (*p* < 0.05) observed in the APRP and *T* of F_1_ aphids at the LC_5_ and L C_10_ concentrations. However, the TPRP was significantly higher in both the LC_5_ and LC_10_ of flonicamid (*p* < 0.05) (Table 3).

**Figure 1:**
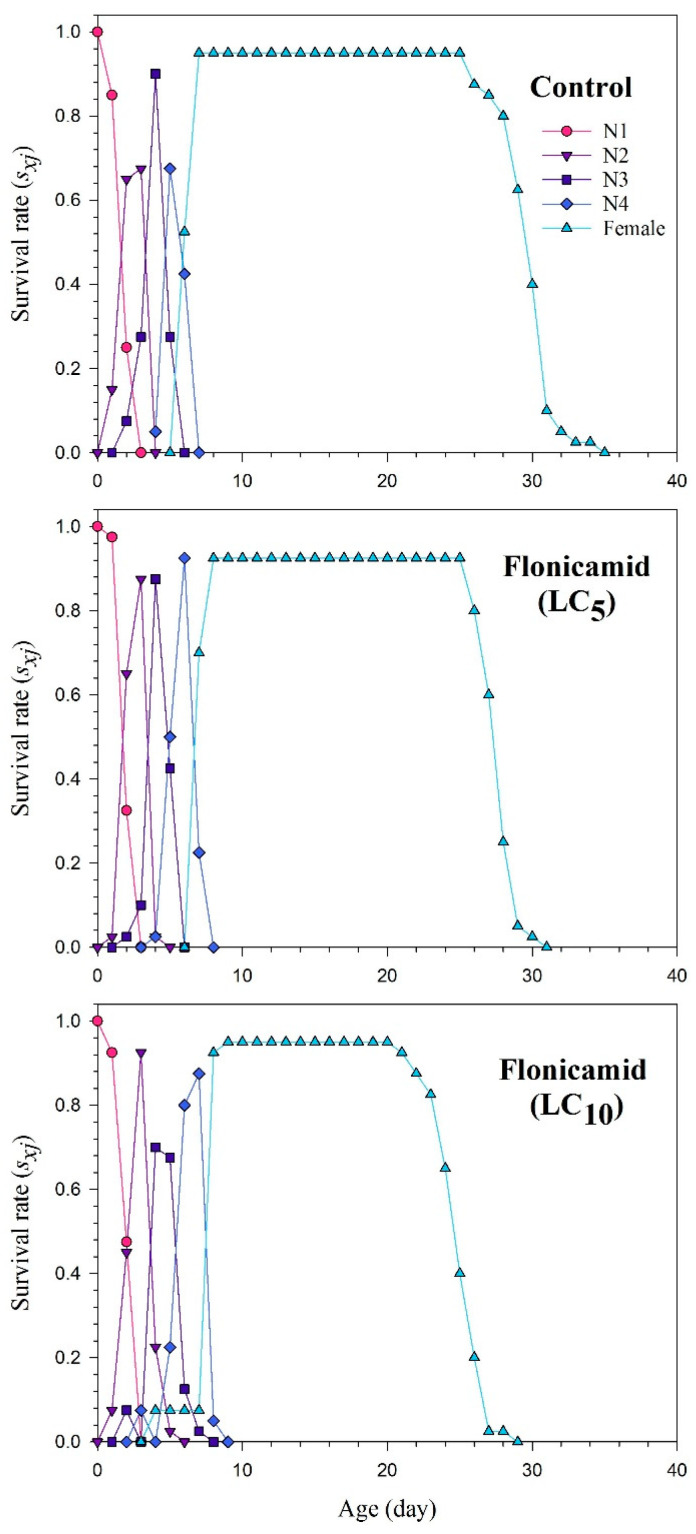
Age-stage-specific survival rate (*s_xj_*) of F_1_ *Aphis gossypii* descended from the F_0_ aphids treated with LC_5_ and LC_10_ of flonicamid and control.

**Figure 2:**
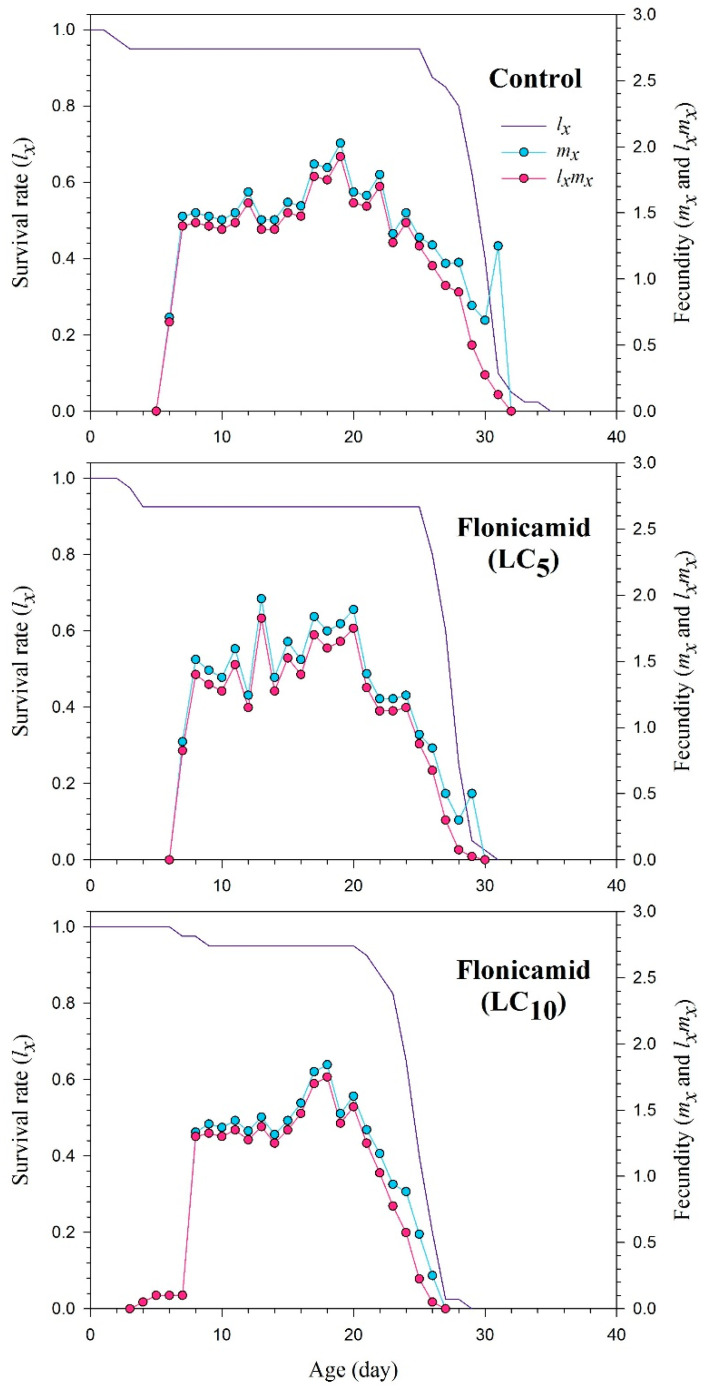
Age-specific survival rate (*l_x_*), age-specific fecundity (*m_x_*), and the age-specific maternity (*l_x_m_x_*) of the F_1_ *Aphis gossypii* descended from the F_0_ aphids treated with LC_5_ and LC_10_ of flonicamid and control.

**Figure 3:**
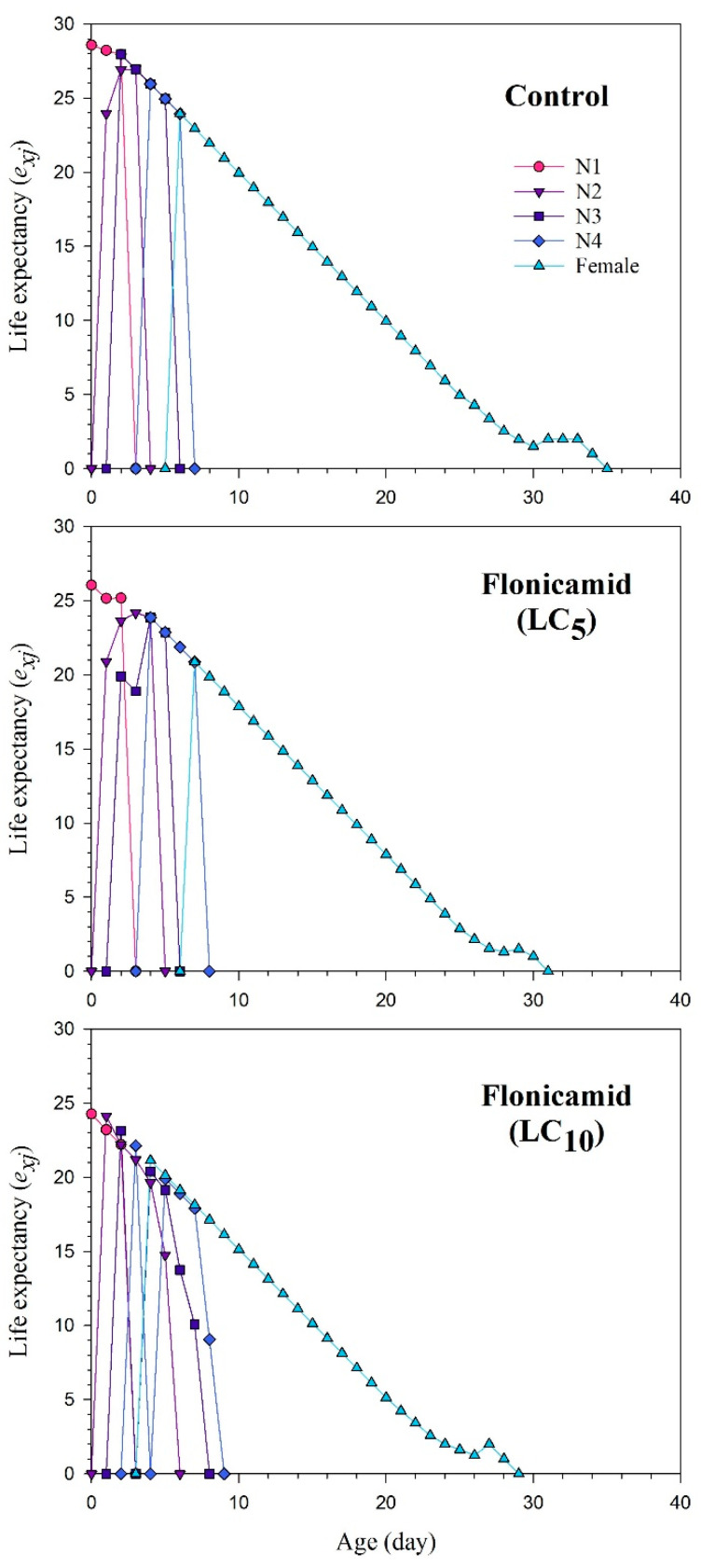
Age-stage life expectancy (*e_xj_*) of F_1_ *Aphis gossypii* descended from the F_0_ aphids treated with LC_5_ and LC_10_ of flonicamid and control.

**Figure 4:**
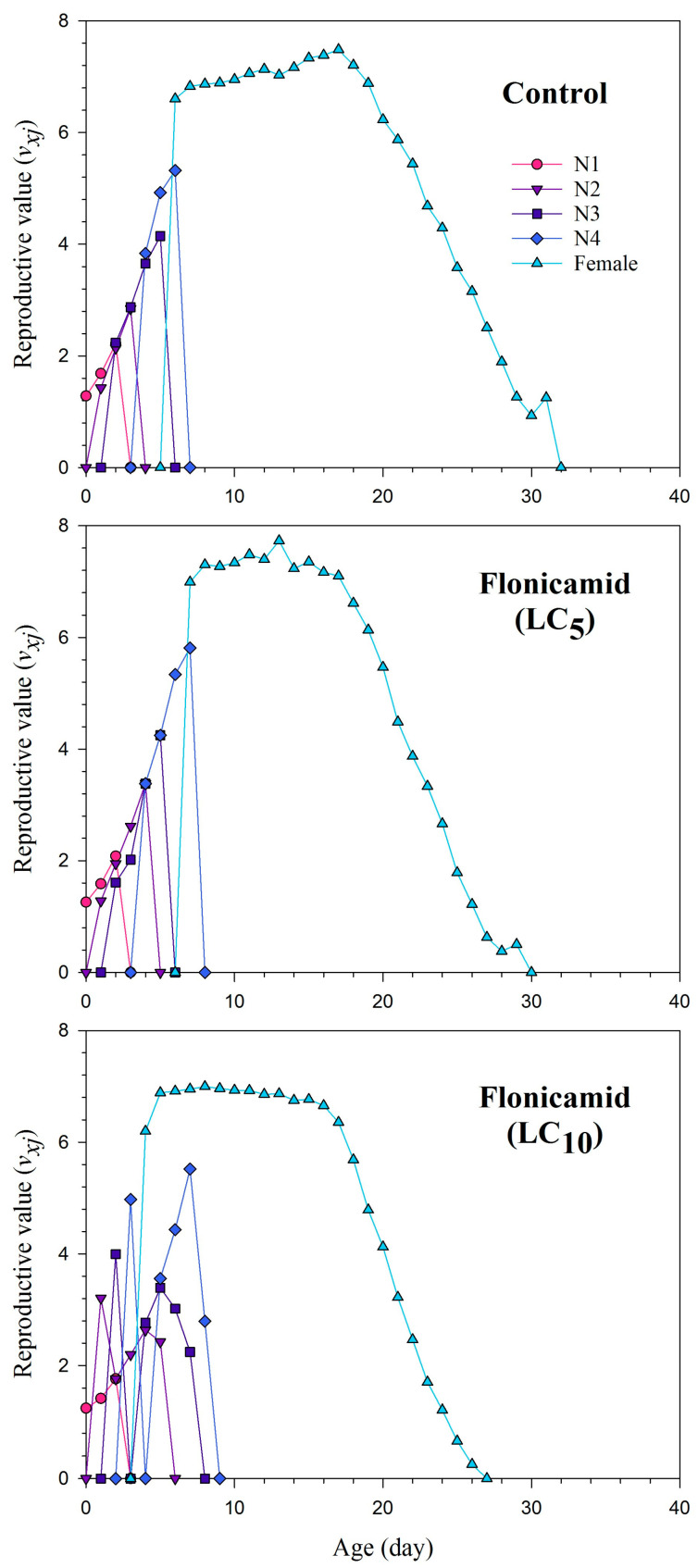
Age-stage reproductive value (*v_xj_*) of F_1_ *Aphis gossypii* descended from the F_0_ aphids treated with LC_5_ and LC_10_ of flonicamid and control.

The *s_xj_* curves show the probability of newly developed *A. gossypii* surviving to age *x* and stage *j* (Figure 1). The plotted curves of *s_xj_* clearly indicate that the sublethal concentrations of flonicamid significantly reduced developmental and adult stages. The impact of the *l_x_*, *m_x_*, and *l_x_m_x_* curves for the flonicamid and control groups of the cotton aphid is shown in Figure 2. The curves of *l_x_*, *m_x_*, and *l_x_m_x_* of *A. gossypii* were substantially affected in the sublethal concentrations of flonicamid as compared to the control (Figure 2). The *exj* curves represent the estimated duration of an individual (*A. gossypii*) at a specific age *x* and stage *j*, indicating the duration of survival beyond age *x* (Figure 3). The F_1_ *A. gossypii* lifespan is probably reduced after the parental generation (F_0_) is exposed to the LC_5_ and LC_10_ concentrations of flonicamid. The *v_xj_* highlights the affection of a population at age *x* and stage *j* towards their prospective progeny Figure 4). The lowest *v_xj_* values were reported in LC_10_, followed by LC_5_, of flonicamid.

### 3.5. Population Projection

**Figure 5:**
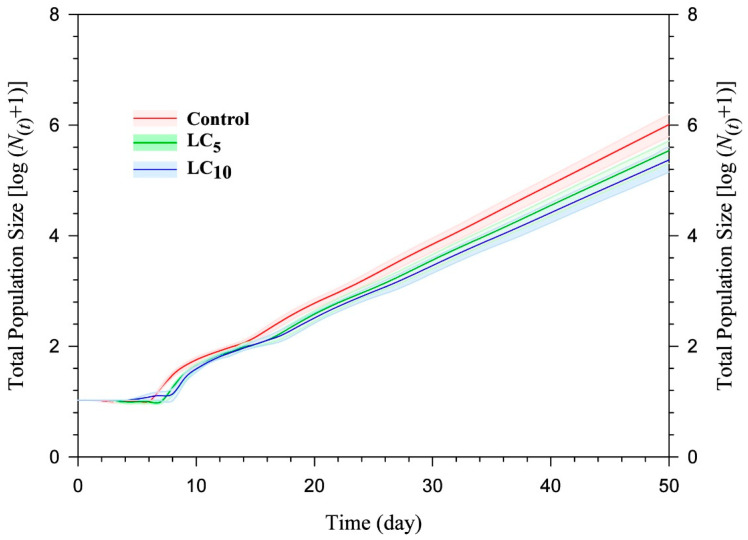
Total population size (*N*_(*t*)_) for F_1_ *Aphis gossypii* descended from the F_0_ aphids treated with LC_5_ and LC_10_ of flonicamid and control (after 50 days) projected from the life table data using the original cohort and the generated cohorts by incorporating the 2.5 and 97.5% percentiles of *R*_0_.

Population projections (50 days) and confidence intervals (2.5th and 97.5th) for the F_1_ *A. gossypii* are shown in Figure 5. Notably, the cohort originating from the untreated control group exhibited the largest total population size of *A. gossypii*, exceeding one million individuals. In contrast, the projections for the F1 progeny whose parents were exposed to flonicamid at LC_5_ and LC_10_ concentrations indicated approximately 350 and 235 thousand aphids, respectively. The total population sizes of *A. gossypii* treated with flonicamid at LC5 and LC10 concentrations were lower than those of the untreated control group after 50 days (Figure 5).

## 4. Discussion

In the current study, we examined the intergenerational sublethal effects of flonicamid on two subsequent generations (F_0_ and F_1_) of *A. gossypii.* The 48 h bioassay results indicated the high toxicity of flonicamid against adult cotton aphids. Several studies reported that sublethal concentrations of pesticides significantly affect the physiological and behavioral traits of exposed insects [14,26,27,28,50]. Hence, it is essential to examine the intergenerational sublethal effects of flonicamid on the biological and demographic parameters of *A. gossypii,* which might be useful for controlling this major pest. The results of this study show that adult *A. gossypii* (F_0_) had reduced longevity and fecundity after being exposed to sublethal concentrations of flonicamid for 48 h.

Our results are in line with a previous study that the longevity and fecundity of F_0_ *Schizaphis graminum* (Rondani) were substantially reduced following exposure to the sublethal concentrations of flonicamid [12]. Similar reduced fecundity was observed by Shi et al. (2022) when they exposed *A. gossypii* to the sublethal and low lethal concentrations of flonicamid [8]. Afidopyropen at sublethal concentrations greatly reduced the longevity and fecundity of F_0_ *A. gossypii* [51]. Also, *A. gossypii* experienced a reduction in fecundity and longevity when directly exposed to the LC_5_ and LC_15_ of imidacloprid [18]. The negative consequences, such as a decreased fertility rate and a shorter lifespan, were observed in *M. persicae*, when subjected to the sublethal concentrations of flupyradifurone [52]. The overall lifespan and reproductive capabilities of *S. graminum* experienced a substantial drop when exposed to sublethal concentrations of acetamiprid [53]. Furthermore, Cui et al. (2018) observed a decrease in longevity and fertility in parental *A. gossypii* after treatment with sublethal concentrations of cycloxaprid [54]. These findings demonstrated that adult longevity and fecundity were negatively affected when insects survived after exposure to the insecticide residues.

The developmental stages of F_1_ *A. gossypii* were considerably affected by the exposure of the parental aphids (F_0_) to the sublethal concentrations of flonicamid. The results indicated increased preadult stages of F_1_ *A. gossypii* at sublethal concentrations of flonicamid. The flonicamid markedly decreased the adult longevity and total longevity of F_1_ aphids. These findings indicated that the developmental stages and overall lifespan of *A. gossypii* were adversely affected by sublethal concentrations of flonicamid. Shi et al. (2021) documented the prolonged developmental duration of 1st instar *A. gossypii* at sublethal and low lethal concentrations of flonicamid [8]. Recently, Gul et al. (2023) reported that the LC_5_ and LC_10_ of flonicamid significantly increased the preadult stages while reducing the adult longevity of *S. graminum* [12]. The increased preadult stages and shorter longevity of F_1_ *A. gossypii* were noted at sublethal concentrations of afidopyropen [51]. Extended developmental durations of F_1_ *A. gossypii* were also reported for thiamethoxam and methyl benzoate treatments [19,55]. However, several studies have documented a reduction in the developmental durations of F_1_ individuals when the F_0_ was treated with insecticides [19,52,56]. The F_1_ *A. gossypii* had decreased longevity when the F_0_ generation was exposed to clothianidin, flupyradifurone, and buprofezin [16,57,58]. Buprofezin and abamectin at low lethal concentrations affect the longevity of *Sogatella furcifera* Horváth (Hemiptera: Delphacidae) and *Scolothrips longicornis* Priesner (Thysanoptera: Thripidae) [59,60]. Prolonged developmental stages and reduced longevity did not indicate the hormetic effects of flonicamid on progeny aphids (F_1_) after exposure of F_0_ individuals to sublethal concentrations. This trade-off occurs when insects allocate energy towards detoxifying chemical pesticides, enabling their survival but at the expense of their development [61,62,63,64].

The key demographic traits of F_1_ aphids significantly decreased at sublethal concentrations of flonicamid, suggesting that flonicamid may have potential value as an insecticide even at sublethal concentrations. Significantly decreased biological traits such as *R_0_*, *r*, *λ*, and fecundity were observed in the F_1_ *A. gossypii*, while the TPRP was substantially prolonged compared to the control. Similar decreased key demographic parameters such as fecundity, *R_0_*, *r*, and *λ* were noted when *S. graminum* were exposed to sublethal concentrations of flonicamid [50]. Ma et al. (2022) reported that the fecundity, *r*, and *λ*, were significantly decreased at the LC_10_ of afidopyropen [51]. Sulfoxaflor, imidacloprid, and clothianidin negatively affected the biological traits of *S. graminum* following treatment with sublethal and low lethal concentrations [65]. Koo et al. (2015) reported reduced *R_0_*, longevity, and fecundity in *A. gossypii* at low lethal concentrations of flonicamid [66]. The LC_25_ of sulfoxaflor dramatically reduced the population growth of *A. gossypii* [67]. Reduced fecundity in *A. gossypii* was observed when treated with sublethal concentrations of spirotetramat [68]. Yuan et al. (2017) also showed similar results in which LC_10_ of cycloxaprid resulted in a notable decrease in the lifespan and reproductive capabilities of F_0_ and F_1_ generation aphids [56]. Non-lethal insecticide-induced adverse effects on the life history parameters of individuals could potentially influence population growth [69]. The current study showed that flonicamid, even at sublethal concentrations, affects the directly exposed individuals and the progeny generations owing to intergenerational sublethal effects, which ultimately impact the population growth of *A. gossypii.*

## 5. Conclusions

In general, the current study demonstrated that sublethal concentrations of flonicamid significantly decreased the life-history traits of F_0_ *A. gossypii*. Moreover, we reported that flonicamid induces intergenerational sublethal effects on the F_1_ by impacting the biological and demographic parameters of *A. gossypii*. The obtained results have the potential to be valuable in the development of control strategies for *A. gossypii*. However, additional research is necessary to figure out the long-term sublethal effects of flonicamid on multiple generations under field conditions.

## Figures and Tables

**Table 1:** Impact of sublethal concentrations of flonicamid on the F_0_ generation of *Aphis gossypii*.

Parameters	Control	Flonicamid (LC_5_)	Flonicamid (LC_10_)
	Mean ± SE	Mean ± SE	Mean ± SE
Adult longevity (days)	22.80 ± 0.22 a	19.30 ± 0.20 b	15.80 ± 0.20 c
Fecundity (nymphs/female)	35.10 ± 0.60 a	26.40 ± 0.34 b	21.70 ± 0.38 c
Reproductive days (days)	21.50 ± 0.27 a	17.03 ± 0.23 b	14.05 ± 0.20 c

The means within a row followed by different lowercase letters indicate significant differences among the treatments.

**Table 2:** Effect of sublethal concentrations of flonicamid on the development of F_1_ *Aphis gossypii* descended from F_0_ generations.

Stage		Control		Flonicamid (LC_5_)		Flonicamid (LC_10_)
	*n*	Mean ± SE	*n*	Mean ± SE	*n*	Mean ± SE
First-instar nymph	40	2.10 ± 0.10 b	40	2.30 ± 0.08 ab	40	2.40 ± 0.10 a
Second-instar nymph	38	1.50 ± 0.09 a	38	1.61 ± 0.08 a	40	1.70 ± 0.09 a
Third-instar nymph	38	1.61 ± 0.10 a	37	1.51 ± 0.09 a	39	1.62 ± 0.09 a
Fourth-instar nymph	38	1.21 ± 0.07 c	37	1.81 ± 0.10 b	38	2.11 ± 0.10 a
Pre-adult	38	6.45 ± 0.08 c	37	7.24 ± 0.07 b	38	7.71 ± 0.18 a
Adult (Female)	38	23.50 ± 0.27 a	37	20.62 ± 0.19 b	38	17.42 ± 0.20 c
Total longevity (Female)	38	29.95 ± 0.30 a	37	27.86 ± 0.19 b	38	25.13 ± 0.26 c

The means within a row followed by different lowercase letters indicate significant differences among the treatments (Paired bootstrap test, *p* < 0.05).

**Table 3:** Population parameters of F_1_ *Aphis gossypii* descended from the F_0_ generation exposed to flonicamid.

Parameters ^a^	Control	Flonicamid (LC_5_)	Flonicamid (LC_10_)
	Mean ± SE	Mean ± SE	Mean ± SE
*R*_0_ (offspring/individual)	33.08 ± 1.32 a	26.83 ± 1.33 b	22.63 ± 0.91 c
*r* (day^−1^)	0.2494 ± 0.0044 a	0.2281 ± 0.0045 b	0.2194 ± 0.0050 b
*λ* (day^−1^)	1.2832 ± 0.0057 a	1.2562 ± 0.0057 b	1.2453 ± 0.0062 b
*T* (days)	14.03 ± 0.16 a	14.42 ± 0.14 a	14.22 ± 0.25 a
*F* (nymphs/female)	34.82 ± 0.59 a	29.00 ± 0.61 b	23.82 ± 0.41 c
RP*_d_* (days)	22.05 ± 0.30 a	18.65 ± 0.21 b	16.00 ± 0.25 c
APRP (days)	0.13 ± 0.05 a	0.19 ± 0.07 a	0.11 ± 0.05 a
TPRP (days)	6.58 ± 0.09 b	7.43 ± 0.10 a	7.82 ± 0.17 a

The means within a row followed by different lowercase letters indicate significant differences among the treatments (Paired bootstrap test, *p* < 0.05). ^a^ R_0_ = *net reproductive rate*; r = *intrinsic rate of increase*; λ = *finite rate of increase*; T = *mean generation time*; F = *fecundity*; *RP_d_* = *reproductive days*; APRP = *adult prereproductive period*; TPRP *= total prereproductive period*.

## Data Availability

All data analyzed during this study are included in this published article.

## Supplementary Materials

The following supporting information can be downloaded at: https://www.mdpi.com/article/10.3390/insects15070529/s1, References [70,71,72,73] are cited in the supplementary materials.
