# Supplementary material for: Intergenerational Sublethal Effects of Flonicamid on Cotton Aphid, Aphis gossypii: An Age-Stage, Two-Sex Life Table Study
Source: Insects. 2024 Jul 13;15(7):529. doi: 10.3390/insects15070529 (PMC11277007; doi:10.3390/insects15070529)
Supplement: Supplementary file 1 [file insects-15-00529-s001.zip › insects-3075734-supplementary.pdf]

Supplementary materials

Intergenerational sublethal effects of flonicamid on cotton aphid, *Aphis gossypii*: An age-stage, two-sex life table study

Hina Gul [1], Ali Güncan [2], Farman Ullah [3], Nicolas Desneux [4,*], Xiaoxia Liu [1,*]

[1] MARA Key Laboratory of Pest Monitoring and Green Management, Department of Entomology, College of Plant Protection, China Agricultural University, Beijing 100193, China; gulhina680@gmail.com (HG); liuxiaoxia611@cau.edu.cn (XL)

[2] Department of Plant Protection, Faculty of Agriculture, Ordu University, 52200, Ordu, Turkey; guncan.ali@gmail.com (AG)

[3] State Key Laboratory for Managing Biotic and Chemical Threats to the Quality and Safety of Agro-Products, Institute of Plant Protection and Microbiology, Zhejiang Academy of Agricultural Sciences, Hangzhou 310021, China; farmanullah787@gmail.com (FU)

[4] Université Côte d'Azur, INRAE, CNRS, UMR ISA, 06000 Nice, France; nicolas.desneux@inrae.fr (ND)

ORCID for Hina Gul: https://orcid.org/0000-0003-1216-7839

ORCID for Ali Güncan: https://orcid.org/0000-0003-1765-648X

ORCID for Farman Ullah: https://orcid.org/0000-0001-6174-1425

* Corresponding authors: nicolas.desneux@inrae.fr (ND); liuxiaoxia611@cau.edu.cn (XL)

Life Table Analysis

The l_x and m_x were determined using Eq. 1 and 2:

$$l_x = \sum_{j=1}^{\beta} s_{xj} \qquad (1)$$

$$m_x = \frac{\sum_{j=1}^{\beta} s_{xj} f_{xj}}{\sum_{j=1}^{k} s_{xj}} \qquad (2)$$

where s_{xj} represents the possibility that a newly born nymph will survive to age x and stage j. β shows number of stages, while f_{xj} represents age–stage-specific fecundity of the individual at age x and stage j.

The RP_d shows days of reproduction and was estimated using Eq. 3:

$$\mathrm{RP}_d = \frac{\sum_{x=1}^{N_f} D_x}{N_f} \qquad (3)$$

where N_f represents number of female adults and D_x shows number of days that a female produced offspring [1].

r shows population growth rate when the time reaches infinity, and the population achieves a stable age–stage distribution. The insect's population might be increased at a rate per unit of time. The r was calculated using the interactive bisection method and corrected with the Euler–Lotka equation with age indexed from 0 [2]:

$$\sum_{x=0}^{\infty} e^{-r(x+1)} l_x m_x = 1 \qquad (4)$$

λ represents population growth rate when time reaches infinity and population attains stable age–stage distribution. The population size will increase at the rate of λ per time unit. The λ was estimated using Eq. 5:

$$\lambda = e^r \qquad (5)$$

R_0 indicates the total number of nymphs laid by a single female until death. The R_0 was calculated using Eq. 6:

$$R_0 = \sum_{x=0}^{\infty} l_x m_x \tag{6}$$

T represents the time required for a population to increase to R_0-fold its current size at a stable rate of increase. The T was calculated using Eq. 7:

$$T = \frac{\ln R_0}{r} \tag{7}$$

e_{xj} shows the predicted duration that an individual of age x and stage j will survive. The e_{xj} was calculated according to Chi and Su [3] using Eq. 8:

$$e_{xj} = \sum_{i=x}^{\infty} \sum_{y=j}^{\beta} s'_{iy} \tag{8}$$

where s'_{iy} indicates the probability that an individual aphid of age x and stage j will survive to age i and stage y by assuming $s' = 1$.

v_{xj} shows the dedication to future offspring at age x and stage j. The v_{xj} was calculated using Eq. 9 according to [4]

$$v_{xj} = \frac{e^{r(x+1)}}{s_{xj}} \sum_{i=x}^{\infty} e^{-r(i+1)} \sum_{y=j}^{\beta} s'_{iy} f_{iy} \tag{9}$$

References

1. Chen, G.-M.; Chi, H.; Wang, R.-C.; Wang, Y.-P.; Xu, Y.-Y.; Li, X.-D.; Yin, P.; Zheng, F.-Q. Demography and uncertainty of population growth of *Conogethes punctiferalis* (Lepidoptera: Crambidae) reared on five host plants with discussion on some life history statistics. *J Econ Entomol* **2018**, *111*, 2143-2152.
2. Goodman, D. Optimal life histories, optimal notation, and the value of reproductive value. *The Am Nat* **1982**, *119*, 803-823.
3. Chi, H.; Su, H.-Y. Age-stage, two-sex life tables of *Aphidius gifuensis* (Ashmead)(Hymenoptera: Braconidae) and its host *Myzus persicae* (Sulzer)(Homoptera: Aphididae) with mathematical proof of the relationship between female fecundity and the net reproductive rate. *Environ Entomol* **2006**, *35*, 10-21.
4. Tuan, S.J.; Lee, C.C.; Chi, H. Population and damage projection of *Spodoptera litura* (F.) on peanuts (*Arachis hypogaea* L.) under different conditions using the age-stage, two-sex life table. *Pest Manag Sci* **2014**, *70*, 805-813.
